# Inhibition of p38 Mitogen-activated Protein Kinase Impairs Influenza Virus-induced Primary and Secondary Host Gene Responses and Protects Mice from Lethal H5N1 Infection*[Fn FN2]

**DOI:** 10.1074/jbc.M113.469239

**Published:** 2013-11-04

**Authors:** Yvonne Börgeling, Mirco Schmolke, Dorothee Viemann, Carolin Nordhoff, Johannes Roth, Stephan Ludwig

**Affiliations:** From the ‡ Institute of Molecular Virology, Center for Molecular Biology of Inflammation,; § Interdisciplinary Center for Clinical Research,; ‖ Cells in Motion Cluster of Excellence, and; ¶ Institute of Immunology, University of Muenster, Muenster 48149, Germany

**Keywords:** Endothelium, Influenza Virus, Interferon, p38 MAPK, STAT Transcription Factor, JAK/STAT Signaling, Highly Pathogenic Avian Influenza Virus (HPAIV), Hypercytokinemia

## Abstract

Highly pathogenic avian influenza viruses (HPAIV) induce severe inflammation in poultry and men. One characteristic of HPAIV infections is the induction of a cytokine burst that strongly contributes to viral pathogenicity. This cell-intrinsic hypercytokinemia seems to involve hyperinduction of p38 mitogen-activated protein kinase. Here we investigate the role of p38 MAPK signaling in the antiviral response against HPAIV in mice as well as in human endothelial cells, the latter being a primary source of cytokines during systemic infections. Global gene expression profiling of HPAIV-infected endothelial cells in the presence of the p38-specific inhibitor SB 202190 revealed that inhibition of p38 MAPK leads to reduced expression of IFNβ and other cytokines after H5N1 and H7N7 infection. More than 90% of all virus-induced genes were either partially or fully dependent on p38 signaling. Moreover, promoter analysis confirmed a direct impact of p38 on the IFNβ promoter activity. Furthermore, upon treatment with IFN or conditioned media from HPAIV-infected cells, p38 controls interferon-stimulated gene expression by coregulating STAT1 by phosphorylation at serine 727. *In vivo* inhibition of p38 MAPK greatly diminishes virus-induced cytokine expression concomitant with reduced viral titers, thereby protecting mice from lethal infection. These observations show that p38 MAPK acts on two levels of the antiviral IFN response. Initially the kinase regulates IFN induction and, at a later stage, p38 controls IFN signaling and thereby expression of IFN-stimulated genes. Thus, inhibition of MAP kinase p38 may be an antiviral strategy that protects mice from lethal influenza by suppressing excessive cytokine expression.

## Teaser

**Background:** Early cytokine dysregulation upon infection with highly pathogenic avian influenza viruses (HPAIV) is a major determinant of viral pathogenicity.

**Results:** p38 MAPK controls HPAIV-induced gene expression by regulating interferon synthesis and subsequently interferon signaling, whereas its inhibition protects mice from lethal infection.

**Conclusion:** p38 MAPK is crucial for the induction of hypercytokinemia upon infection.

**Significance:** Targeting p38 MAPK is a promising approach for antiviral intervention.

## Introduction

Systemic infection of humans and birds with highly pathogenic avian influenza viruses (HPAIV)^4^ of the H5N1 subtype is characterized by severe internal bleeding, multiorgan failure, and hyperreaction of the host immune response that leads to massive overproduction of cytokines and chemokines known as the “cytokine storm.” There is evidence that the latter contributes to the pathogenesis of human H5N1 disease (1–3), but the source of this cell-intrinsic phenomenon is still under investigation. So far, neuro- and endothelial cell tropism are known to play a role in HPAIV infections (4, 5) and, recently, specific response patterns upon H5N1 infection of endothelial cells (ECs) have been shown to play a crucial role in overwhelming proinflammatory responses compared with infections with low pathogenic strains. This induction of proinflammatory and antiviral genes is strongly regulated by the nuclear factor κ-light chain enhancer of activated B-cells (NF-κB) and is specifically modulated by transcriptional regulators HMGA1 (high-mobility group protein HMG-I/HMG-Y) and NFATC4 (nuclear factor of activated T-cells, cytoplasmic 4) (6, 7). In contrast, Hui and colleagues (8) showed that the cytokine response in primary human macrophages mainly depends on IRF3 (interferon regulatory factor 3) and activator protein 1 (AP1) signaling. However, innate immune cell recruitment and early innate cytokine and chemokine production in the lungs of infected mice have been shown to be independent events, and are both regulated by the pulmonary endothelium (9). The blockade of inflammatory signaling in these cells drastically reduces the immune pathologic effects of HPAIV infection in mice. Therefore, it is very likely that endothelial cells are the main modulators of the HPAIV-induced cytokine storm, whereas lung epithelial cells and inflammatory infiltrates are currently thought to be central for cytokine dysregulation (10).

Cells respond to virus infection by launching a broadly reactive antiviral program, mainly orchestrated by the key cytokine interferon β (IFNβ). It has been shown that the initial induction of IFNβ transcription depends mainly on the same three transcription factors that are thought to be crucial for the cytokine storm: IRF3, NF-κB, and AP1 (6, 8, 11). Upstream of these transcription factors are signaling cascades, which allow the fine regulation of each signaling step within the cascade. Finally, IFNs activate a signal transduction pathway that triggers the transcription of a diverse set of genes that, in total, establish an antiviral response in target cells (12).

Infection with influenza A virus leads to the activation of a variety of intracellular signaling pathways including all four so far known mitogen-activated protein kinase (MAPK) cascades (13). MAP kinases are able to regulate gene expression at both the transcriptional and post-transcriptional levels by different mechanisms, thereby controlling diverse cellular processes (14). Among the different MAP kinase subgroups, a strong link has been established between the p38 pathway and inflammation. It has been postulated that diseases like Alzheimer and inflammatory bowel disease are associated with dysregulation of the p38 pathway (15, 16) and it has been shown that a variety of pathogen- or cell stress-related stimuli can activate p38 MAPK (17, 18). Therefore, the kinase plays an essential role in the production of proinflammatory cytokines such as IL-1β, TNF-α, and IL-6 (19). In recent years, special consideration has been given to the p38 pathway concerning its role in stimulation-specific interferon induction and signaling. Recently, the involvement of p38 MAPK in the HPAIV-mediated dysregulation of cytokine expression in primary human monocyte-derived macrophages and bronchial epithelial cells was hypothesized (8, 20, 21). Furthermore, hyperactivation of p38 and increased cytokine concentrations in plasma samples from patients infected with severe seasonal influenza have been reported (22).

In this study, biochemical as well as genetic tools were used to dissect the role of the p38 pathway in the HPAIV-induced cytokine storm in primary endothelial cells. Global gene expression profiling confirmed that nearly all (94%) HPAIV-induced genes are either partially or fully dependent on this pathway. Further analysis showed that p38 acts not only in the primary induction of cytokines but also affects the secondary cytokine-induced response by modulating the JAK-STAT pathway. Moreover, this study provides evidence for the first time that inhibition of p38 MAPK significantly protects mice from lethal influenza by reducing cytokine-induced pathogenicity. Therefore, interference with the p38 MAPK pathway might be a new target for therapeutic intervention in HPAIV infection.

## EXPERIMENTAL PROCEDURES

### 

#### 

##### Ethics Statement

All animal studies were performed in compliance with animal welfare regulations of the German Society for Laboratory Animal Science (GV-SOLAS) and the European Health Law of the Federation of Laboratory Animal Science Associations (FELASA). The protocol was approved by the State Agency for Nature, Environment and Consumer Protection (LANUV), Germany (permission number Az 8.87–50.10.36.09.007).

##### Viruses and Cells

A/Thailand/KAN-1/2004 (H5N1) was used with kind permission from P. Puthavathana (Bangkok, Thailand). A/FPV/79/Bratislava (H7N7, fowl plague virus) was originally obtained from the Institute of Virology in Giessen, Germany. Viruses were propagated on Madin-Darby canine kidney (MDCKII) cells cultured in minimal essential medium (PAA Laboratories) containing 10% (v/v) FCS (Invitrogen) as described elsewhere (23). Human alveolar epithelial cells (A549), green monkey epithelial cells (Vero), and Phoenix packaging cells (Orbigen) were cultured in DMEM (PAA Laboratories) containing 10% FCS. Primary human umbilical vein endothelial cells (HUVEC; Promocell) were cultured in Endothelial Cell Growth Medium with Supplement Mix (Promocell) and were used at passages three to five.

##### Mouse Experiments

BALB/c mice were obtained from the Harlan-Winkelmann animal breeding facilities. Eight- to 10-week-old mice were anesthetized by intraperitoneal injection of 200 μl of solution of 0.5% ketamine (Ceva) and 0.1% xylazine (Ceva) in PBS. Mice were infected or stimulated by the intranasal route in a 50-μl volume as indicated. Health status of the animals was monitored daily. In agreement with animal welfare regulations, mice were killed upon a body weight loss of 25%. Mouse survival curves are represented by Kaplan-Meier analysis.

##### Reagents and Plasmids

Cells were preincubated with different concentrations of the p38-specific inhibitors SB 202190 or SB 203580 (DMSO soluble, Calbiochem) for 30 min at 37 °C before infection or stimulation as indicated. BALB/c mice were treated intraperitoneally with 20 mg/kg/day SB 202190 hydrochloride (Synkinase) or SB 203580 hydrochloride (Axon). These pyridinyl-imidazol components specifically inhibit p38α and -β by competing with ATP for the same binding site.

Recombinant human IFNβ and -γ were obtained from the PBL Interferon Source and used in concentrations from 100 to 500 units/ml as indicated. The double-stranded RNA analog poly(I:C) was purchased from Amersham Biosciences. Mice were stimulated with 1 μg of poly(I:C) in 50 μl of PBS via the intranasal route.

The retroviral expression plasmid pCFG5-IEGZ HA was previously described (6). The expression plasmid pRC/CMV STAT1α Y701F was obtained from Addgene, provided by J. Darnell (Laboratory of Molecular Cell Biology, Rockefeller University, New York) and previously described (24). The open reading frame from STAT1 Y701F was cloned into pCFG5-IEGZ HA and the double mutant (Y701F/S727A) was obtained by site-directed mutagenesis. Primer sequences are included in supplemental Table S1. The dominant-negative MKK6 mutant expressing vector pCFG5-IEGZ MKK6A was a kind gift from E. Serfling (Institute of Pathology, Wuerzburg). The luciferase reporter construct pTATA-IFNβ-luc was a kind gift from J. Hiscott (Lady Davis Institute, Montréal, Canada) and contains the whole IFNβ promoter upstream of the luciferase gene. Reporter gene constructs pTA-ISRE-luc and pTA-GAS-luc were obtained from Clontech. Upstream of the luciferase gene, pTA-ISRE contains five copies of the ISRE-binding sequence and pTA-GAS-luc two copies of the STAT1 enhancer element.

##### Gene Knockdown by siRNA

Human *MAPK14* siRNA (5′-CAGUCCAUCAUUCAUGCGAAA-3′), human *MAPK11* siRNA (5′-GCCCUGAGGUUCUGGCAAA-3′) as well as control siRNA (5′-UUCUCCGAACGUGUCACGU-3′) were synthesized by MWG-Biotech AG. Transfection of A549 cells was performed with Lipofectamine 2000 (Invitrogen), Vero cells were transfected by the use of HiPerFect (Qiagen), and HUVEC with Oligofectamine (Invitrogen) according to the manufacturer's protocols.

##### Plaque Titration

Plaque forming units of a given virus suspension were determined by a standard plaque assay as described earlier (25). Mouse lung titers were analyzed at the times indicated. Lungs were collected and placed in PBS with Collagenase A (0.7 mg/ml; Roche) to obtain a 10% tissue homogenate and incubated for 90 min at 37 °C. Next, the samples were homogenized by passing them through a 20-gauge needle (0.5 mm diameter) and centrifuged at 10,000 × *g* for 5 min. Supernatants were taken for plaque titration.

##### Retroviral Gene Transfer

The empty retroviral vector pCFG-IEGZ HA or pCFG5-IEGZ STAT1/MKK6 expressing the different phospho-mutants were transfected in Phoenix packaging cells (Orbigen) with polyethyleneimine, selected with 250 μg/ml Zeocin (Invitrogen), and retrovirus-containing supernatants were harvested and used for infection of A549 cells as previously described (6). Retrovirally transduced A549 cells were selected with 250 μg/ml Zeocin for 2 weeks to obtain stable cell lines and the efficiency of retroviral gene transfer was measured by flow cytometric detection of recombinant enhanced GFP (EGFP), which was coexpressed with the gene of interest, using a FACSCalibur cytometer (BD Biosciences) 48 h after transduction. Transduction rates ranged from 90 to 100% and stable STAT1 mutant-expressing cells were subcloned to obtain equal expression levels of the transgenes as measured by Western blot.

##### Western Blot

Cells were lysed in radioimmunoprecipitation assay (RIPA) buffer containing protease and phosphatase inhibitors (25). RIPA protein lysates were cleared by centrifugation, mixed with 5× Laemmli buffer, separated by SDS-PAGE, and blotted onto nitrocellulose membranes. Antisera directed against p38 (C-20), phospho-p38 (12F8), and phospho-STAT1 Ser^727^ were purchased from Cell Signaling Technology. STAT1 (N terminus) and phospho-STAT1 Tyr^701^ (clone 14) antibodies were obtained from BD Transduction Laboratories, Influenza A PB1 (VK-20) and ERK2 (C-14) antibodies were from Santa Cruz Biotechnology. Antiserum against viral PB2 protein was a kind gift from Dr. E. Fodor (Sir William Dunn School of Pathology, Oxford, UK (26)) and Influenza A M1 antibody was purchased from AbD Serotec.

##### RNA Isolation, cDNA Synthesis, and qRT-PCR

Total RNA from cells was isolated using the RNeasy Kit (Qiagen) according to the manufacturer's instructions. Lungs from mice were collected at the time points indicated and total RNA was isolated using TRIzol® reagent (Invitrogen). TRIzol lysis was performed according to the manufacturer's protocol, introducing a secondary phase separation step. Samples were homogenized using a FastPrep-24 homogenizator (MP Biomedicals) with Lysing Matrix D (MP Biomedicals). Isopropyl alcohol-precipitated RNA was dissolved in 0.3 m NaAc (pH 5.2) and phenol (pH 4.1–5.6) was added in a ratio of 1:1 (v/v). After vortexing and centrifugation (4 °C, 5 min, 13,000 × *g*), chloroform was added to the RNA-containing upper phase (1:1, v/v) and again vortexed and centrifuged (4 °C, 5 min, 13,000 × *g*). Subsequently, RNA was precipitated by adding 96% EtOH (1:3, v/v) to the upper phase and followed by washing.

Three micrograms of total RNA were reverse transcribed with Revert AID H Minus Reverse Transcriptase (MBI Fermentas) and oligo(dT) primers according to the manufacturer's protocol. The cDNA was used for qRT-PCR, which was performed using a Roche LightCycler 480 and Brilliant SYBR Green Mastermix (Agilent) according to the manufacturer's instructions. Primer sequences are included in supplemental Table S1. Relative changes in expression levels (*n*-fold) were calculated according to the 2^−ΔΔ^*^CT^* method (27).

##### Luciferase Assay

Transfection of Vero or A549 cells with different luciferase reporter plasmids (0.3 μg) was performed with Lipofectamine 2000 (Invitrogen) according to the manufacturer's instructions. Luciferase assays were carried out 24 h post-transfection as previously described (28). Relative light units were normalized to protein concentrations determined with a standard Bradford assay.

##### DNA Microarray and Statistical Data Analysis

Primary HUVEC were treated with 20 μm SB 202190 or DMSO for 30 min at 37 °C and subsequently infected with FPV for 5 h with a multiplicity of infection (m.o.i.) of 5 or left uninfected. Total RNA was isolated from three independent experiments using the RNeasy kit (Qiagen). Samples were processed for microarray hybridization using Affymetrix Human Genome 133 Plus 2.0 Gene Arrays according to the manufacturer's protocol. The GeneChip Scanner 3000 detected fluorescent signals were recorded and computed by Affimetrix GeneChip Operating Software version 1.4. Parts of the data set concerning FPV-infected HUVEC and control HUVEC have also been used in a previous study by our group (7).

For a more elaborate data analysis, the Expressionist Suite software package from GeneData (Basel, Switzerland) was used as previously described (29). Only genes with a fold-change (FC) of >2.0 or <2.0 and *p* ≤ 0.05 (paired *t* test) of three independent experiments were considered as regulated. “On/off”-regulated genes were evaluated as described (29), considering genes with on/off ratios of 0:3, 0:2, 1:3, 3:0, 2:0, and 3:1, respectively. From this group, only regulation with a high FC of ≥5 and *p* < 0.05 were included in the list of regulated genes to differentiate on/off phenomena occurring around the background threshold. Principle component analysis was applied to mathematically reduce the dimensionality of the entire spectrum of gene expression values of the microarray experiment to three components (30).

By definition, strictly p38-dependent genes were either fully switched off or reduced below threshold in the presence of SB 202190. By this mathematical method the strength of mRNA induction as well as constitutive expression on a high level may not be reflected to calculate strict *versus* partial dependence. Therefore, the distinction might be to some extent arbitrarily.

To identify functional categories of genes that are overrepresented in the data sets of regulated genes, Gene Ontology (GO) annotations to every probe set spotted on the Affymetrix 133 Plus 2.0 Array were assigned and compared with the distribution of GO annotations in the gene group of interest by applying the Fisher exact test. In the case of genes that were represented by two or more probe sets, only one transcript was taken into account to avoid potential bias.

##### Statistical Analysis

Statistical significance between samples was determined using unpaired Student's *t* test. Values of *, *p* < 0.05; **, *p* < 0.01; and ***, *p* < 0.001 are indicated.

## RESULTS

### 

#### 

##### p38 MAPK Is Activated Upon Influenza A Virus Infection in Endothelial Cells

**FIGURE 1.**
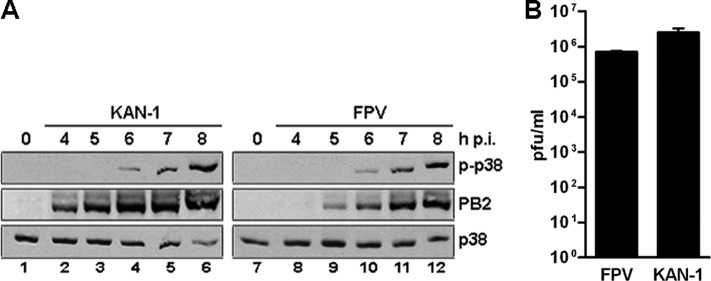
**p38 is activated upon influenza A virus infection in endothelial cells.** *A,* Western blot analysis of total lysates of HUVEC infected with 5 m.o.i. of different influenza viruses of subtypes H7N7 (*FPV*) or H5N1 (*KAN-1*). P-p38 was detected 4–8 h post-infection (*h p.i.*) (*upper panels*). Efficient infection was confirmed by immunostaining for viral PB2 protein (*middle panels*). Equal loading was verified by the detection of total p38 (*lower panels*). Blots are representative of three independent experiments. *B,* comparison of viral replication abilities of different influenza isolates. HUVEC were preincubated with 20 μm SB 202190 or DMSO and subsequently infected with FPV or KAN-1 with 1 m.o.i. for 9 h. Viral titers were determined by a standard plaque assay and are depicted as mean ± S.D. of three independent experiments.

Compared with low pathogenic influenza viruses, HPAIV are unique in inducing a broad spectrum cytokine response, contributing to virus-associated immune pathology. Previously, activation of p38 MAPK was shown to modulate antiviral signaling responses in bronchial epithelial cells and monocyte-derived macrophages upon influenza virus infection (20, 21). Furthermore, differential activation of diverse MAPKs was observed in macrophages upon influenza infection with various subtypes (31). But, so far, nothing is known about the activity patterns of p38 in endothelial cells upon infection with diverse influenza isolates. Therefore, primary HUVEC were infected with two highly pathogenic subtypes of human or avian origin, A/Thailand/KAN-1/2004 (H5N1) or A/FPV/Bratislava/79 (H7N7). Both influenza isolates activate p38 upon infection, demonstrated by phosphorylation of threonine (Thr^180^) and tyrosine (Tyr^182^) residues in the activation loop of p38 MAPK (Fig. 1*A*, *upper panels*). Activation of p38 was comparable, despite slight differences in viral replication kinetics. Viral polymerase protein PB2 of the H5N1 (KAN-1) strain was detected moderately earlier (Fig. 1*A*, *middle panels*), but the 9-h post-infection titers were not significantly higher compared with the H7N7 (FPV) isolate as determined by standard plaque titration (Fig. 1*B*).

##### p38 MAPK Signaling Has a Major Impact on the HPAIV-induced Gene Profile

**FIGURE 2.**
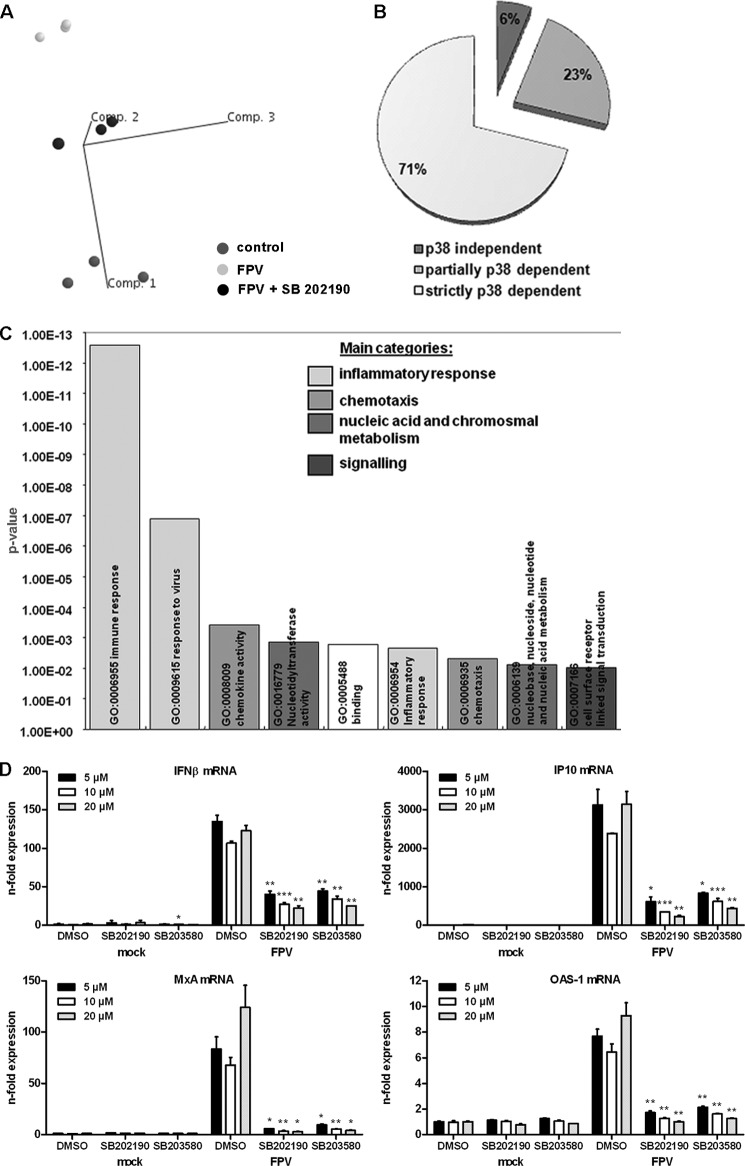
**Influence of p38 inhibition on the HPAIV-induced transcriptome.** HUVEC were preincubated with 20 μm SB 202190 or DMSO. Cells were infected with 5 m.o.i. of FPV and incubated with SB 202190 for 5 h. *A,* principle component analysis displaying gene expression profiles of uninfected HUVEC (*control*) and FPV-infected HUVEC in the presence or absence of SB 202190 of three independent experiments. Vector clouds represent the up-regulated/switched on mRNAs of individual experiments and are positioned in a three-dimensional vector space according to their variance to each other. *B,* Venn diagram displaying the relative distribution of FPV-induced mRNAs (percent) according to the p38 MAPK dependence determined by microarray analysis of three independent experiments. Strictly p38-dependent mRNAs show expression levels below 2-fold in the presence of SB 202190. *C,* clustering of SB-dependent FPV-induced genes (up-regulated and switched on) according to their GO annotated function. Plotted is the statistical significance (*y* axis) of overrepresentation compared with the distribution of functional gene groups on the whole microarray according to Fisher's exact test. Related GO groups are displayed by *identically color bars* and summarized into main categories of overrepresented functional groups (restricted to two genes/GO-group + *p* value <0.01). *D,* HUVEC were incubated with different concentrations (5, 10, and 20 μm) of SB 202190 or SB 203580 in comparison to DMSO and infected with FPV (5 m.o.i.) for 5 h. Expressional changes of mRNAs of different cytokines were detected by qRT-PCR and are depicted as mean *n*-fold (± S.D.) of one representative experiment normalized to control mock (DMSO, 5 μm).

To gain broad insight into what role p38 MAPK might play in the HPAIV-induced cytokine storm, a comparative global gene expression study was performed. HPAIV-induced gene expression was monitored at 5 h post-infection, which is well within the first replication cycle of influenza virus infection and thus minimizes the effects of secondary infection. In this array, primary HUVEC were preincubated with 20 μm SB 202190, a specific p38α/β inhibitor, or left untreated for 30 min and subsequently infected with the HPAIV strain FPV (H7N7). Total RNA of infected and uninfected control HUVEC was processed for microarray hybridization and the data sets from untreated FPV- or mock-infected cells were used for comparison (7). FPV infection led to the up-regulation of 82 mRNAs, including 19 genes being switched on compared with mock-infected control cells. More than 4000 mRNAs were down-regulated or switched off upon H7N7 infection. The final analysis exclusively focused on up-regulated genes because unspecific 5′ cap snatching mechanisms (32) or interference with the processing of cellular RNAs by the viral NS1 protein (33) significantly contribute to the process of gene down-regulation by influenza viruses, which makes the identification of specifically down-regulated genes impossible. To verify the impact of p38 MAPK on the FPV-induced gene profile, principal component analysis was performed, displaying all influenza-inducible genes as vector clouds in a three-dimensional vector space. Here, the consistency of the gene profiles within the same experimental group was confirmed, indicating reliable reproduction of data in the different experiments (Fig. 2*A*). Furthermore, clear separation of the experimental groups could be observed, illustrating that the induced gene expression patterns in these groups were specific and distinct from each other. Comparison of the two different data sets of FPV-infected HUVEC showed that inhibition of p38 led to a partial reversion of the FPV-induced gene spectrum. This result implies that either only a distinct gene subset is strictly dependent on p38 signaling or that there are some genes that show only a slight requirement for the kinase. Indeed, mathematical analysis showed that 71% of FPV-induced genes were strictly dependent (switched off in the presence of SB 202190) and 23% partially dependent on p38 signaling (Fig. 2*B* and supplemental Table S2). Although this distinction might be to some extent arbitrary, more than 90% of the FPV-induced genes were found to be p38 dependent.

By functional clustering according to gene ontology annotations, Viemann and colleagues (7) revealed that the majority of mRNAs induced upon FPV infection belong to the inflammatory viral response and cell-cell signaling categories (7). p38-dependent genes cluster into the immune/inflammatory response and chemotaxis categories, demonstrating that p38 MAPK plays a prominent role in the expression of the major gene groups induced by HPAIV and is thereby crucial for the dysregulation of cytokines and chemokines (Fig. 2*C*). To validate the microarray data, quantitative real-time RT-PCR (qRT-PCR) analysis was performed for a subset of FPV-induced mRNAs in the presence or absence of SB 202190. All tested FPV-induced mRNAs were at least partially down-regulated upon p38 inhibition, as shown in supplemental Fig. S1. To exclude nonspecific off-target effects of the inhibitor in microarray experiments, qRT-PCR analysis was performed for different FPV-induced mRNAs in the presence or absence of different doses of SB 202190 (5, 10, and 20 μm) in comparison with a second p38-specific inhibitor SB 203580 (Fig. 2*D*). As expected, treatment with both inhibitors led to significantly reduced cytokine expression levels in a concentration-dependent manner. These observations reflect the prominent role of p38 MAP kinase in the expression of HPAIV-induced genes.

##### p38 MAPK Activity Is Required for H5N1-induced Expression of IFNs and ISGs

**FIGURE 3.**
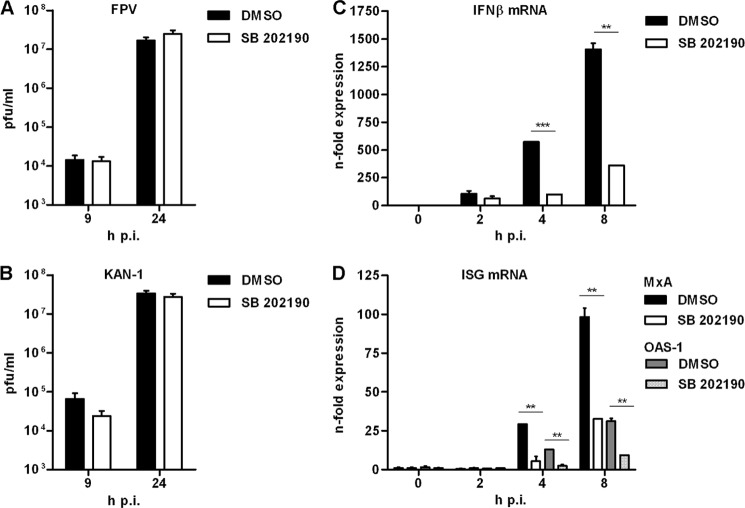
**Time course of H5N1-induced genes in the presence or absence of SB 202190.** *A* and *B,* effects of p38 MAPK inhibition on viral replication. HUVEC were preincubated with 20 μm SB 202190 or DMSO and subsequently infected with FPV (*A*) or KAN-1 (*B*) with 0.01 m.o.i. for the indicated time points. Viral titers were determined by a standard plaque assay and are depicted as mean ± S.D. of three independent experiments. *C* and *D,* HUVEC were preincubated with 20 μm SB 202190 or DMSO. Cells were infected with 5 m.o.i. of KAN-1 and incubated with SB 202190 for the indicated time points. Expressional changes of mRNAs of IFNβ (*C*) and different ISGs (*D*) were detected by qRT-PCR and are depicted as mean *n*-fold (± S.D.) of one representative experiment normalized to control.

In 2009, Nencioni and colleagues (34) described a decrease in viral titers due to the retention of viral ribonucleoproteins in the nucleus when p38 MAPK was inhibited in Madin-Darby canine kidney cells. Furthermore, it was shown that virus internalization is decreased upon p38 inhibition in bronchial epithelial cells (35). To ensure that the observed effects on gene induction were not due to replication differences, the potential influence of SB 202190 on viral propagation of FPV and KAN-1 was assessed in HUVEC by standard plaque assays. The results shown in Fig. 3, *A* and *B,* clearly indicate that the inhibitor did not significantly affect replication efficiency of the two viruses in endothelial cells. Thus, the observed impact of p38 inhibition on gene expression in HUVEC is replication independent and is primarily due to direct interference with the inducing signaling pathways.

To assure that the observed effects of p38 MAPK signaling on the HPAIV-induced gene profile were not specific for the avian isolate FPV but also true for other highly pathogenic isolates of other subtypes, HUVEC were infected in the presence or absence of SB 202190 with the human H5N1 isolate KAN-1. Total RNA was isolated 2, 4, and 8 h post-infection, and qRT-PCR analysis was performed for a subset of KAN-1-induced mRNAs. Because inflammatory and immune genes were mainly affected by p38 inhibition, this study is focused on IFNβ, as a major mediator of the innate antiviral response (Fig. 3*C*) and interferon-stimulated genes (ISGs) (Fig. 3*D*). qRT-PCR data confirmed the dependence of IFNβ and ISG production on p38 MAP kinase signaling in cells infected with either FPV or KAN-1. Furthermore, nonspecific off-target effects of SB 202190 on KAN-1-induced cytokine expression were ruled out in an inhibitor-independent approach by using siRNAs specific for different p38 isoforms, p38α (*MAPK14*) and p38β (*MAPK11*) (supplemental Fig. S2, *A* and *B*). This method also allows to evaluate the role of the two different isoforms, and the results indicate a more prominent function of p38α in cytokine expression upon H5N1 infection especially in the case of IFNβ.

Viral RNA is the main pathogen-associated molecular pattern recognized by different pattern-recognition receptors, inducing the type I IFN response in virus-infected cells. Especially detection of viral 5′-triphosphate RNAs by the cytoplasmic helicase RIG-I (retinoic acid inducible gene I) plays an important role in influenza A virus infection (36). To test if the blockade of p38 MAPK inhibits viral RNA-induced signaling, HUVEC were transfected with RNA from uninfected or HPAIV-infected A549 cells in the presence or absence of SB 202190 for 3 h (supplemental Fig. S2*C*). In contrast to RNA from uninfected cells, stimulation with total RNA from virus-infected cells led to an induction of IFNβ and ISG mRNAs in a p38 MAPK-dependent fashion. These results clearly show that p38 plays an important role in the induction of IFNβ and consequently in the expression of interferon-stimulated genes upon HPAIV infection in endothelial cells, confirming previous results obtained in monocytic cells (8).

##### p38 MAPK Signaling Has a Direct Impact on IFNβ Promoter Activity

p38 MAPK signaling can influence the production of IFNβ and ISGs on different levels. It has recently been shown that there is a stimulation-specific contribution of p38 MAPK to IFNβ gene expression in human macrophages (37) that might be due to the activation of ATF-2 (38) as well as the cross-regulation of NF-κB (39) and IRF3 (40), the major components of the IFNβ enhanceosome. Moreover, p38 MAPK modulates IFN signaling by affecting the JAK/STAT pathway. For instance, p38 can positively regulate JAK/STAT signaling by the phosphorylation of STAT1 at serine 727 (Ser^727^) or by the activation of cytosolic phospholipase A_2_ (41, 42). Modulation of STAT signaling may in turn influence IFN expression as a positive regulatory feedback loop. One of the first ISGs produced in response to IFNβ signaling is interferon regulatory factor 7 (*IRF7*). IRF7 can form homo- or heterodimers with IRF3 and replaces IRF3 in the later stages of IFNβ expression, thereby again regulating IFN expression (12). To address whether p38 MAPK signaling mainly affects the initial production of IFNβ or IFN signaling on the STAT1 level in response to influenza A virus infection, experimental systems assessing both signaling steps were used.

**FIGURE 4.**
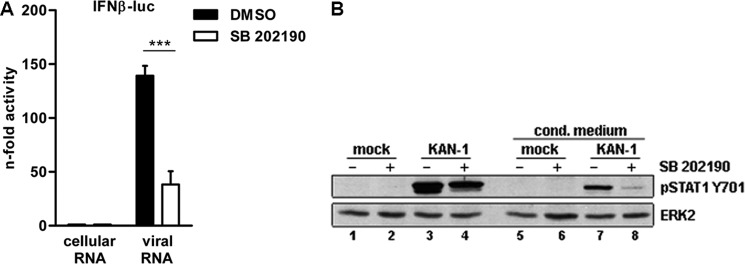
**p38 MAPK inhibition affects H5N1-induced IFN expression.** *A,* impact of p38 MAPK inhibition on the IFNβ promoter activity. Vero cells were transfected with the IFNβ promoter for 24 h. Cells were preincubated with 20 μm SB 202190 or left untreated and subsequently stimulated with 500 ng of total RNA isolated from infected A549 cells (8 h, 5 m.o.i.). Total RNA from uninfected A549 cells was used as control. 5 h p.s. promoter activity was measured by a luciferase assay and the results are depicted as mean *n*-fold (± S.D.) of three independent experiments normalized to controls. *B,* Western blot analysis of total lysates of HUVEC treated with UV-inactivated, filtered conditioned media from mock-infected control cells (*lanes 5* and *7*) and KAN-1-infected cells (5 m.o.i., 5 h) (*lanes 6* and *8*). Donor cells were pretreated with DMSO (*lanes 1* and *3*) or SB 202190 (20 μm, *lanes 2* and *4*). STAT1 Tyr^701^ phosphorylation was detected 15 min after treatment with conditioned medium (*upper panel*). Equal loading was verified by the detection of total ERK2 (*lower panel*). Blots are representative of three independent experiments.

The first indication of a direct impact of p38 MAP kinase on the IFN enhanceosome activity in response to HPAIV infection was provided by the observation that a reduction in IFNβ mRNA levels occurs as early as 2 h post-infection when p38 is inhibited (Fig. 3*C*). To verify that p38 MAPK directly affects the production of IFNβ on the promoter level, Vero cells lacking functional type I IFN genes were transfected with total RNA isolated from HPAIV-infected A549 cells and an IFN reporter plasmid (Fig. 4*A*). Reduction of IFNβ reporter activity in the presence of SB 202190 indicated a direct effect of p38 signaling on IFNβ expression, independent of any auto- or paracrine actions of viral RNA-induced type I IFN. Furthermore, nonspecific off-target effects of the inhibitor were ruled out by using a dominant-negative mitogen-activated protein kinase kinase 6 mutant (MKK6A) acting upstream of p38 MAPK (supplemental Fig. S3*A*).

The release of type I IFNs and other JAK/STAT activating cytokines from influenza virus-infected cells can be monitored by STAT1 phosphorylation on tyrosine at position 701 (Tyr^701^), which is a hallmark of IFN signaling. To explore the impact of p38 MAPK on IFN mRNA production in HPAIV-infected cells on the protein level, conditioned medium experiments were performed with DMSO- or SB 202190-pretreated HUVEC, which were infected with KAN-1 for 5 h (5 m.o.i.). Supernatants were subsequently transferred to untreated HUVEC for 15 min and STAT1 phosphorylation was assessed. Fig. 4*B* shows that the observed STAT1 Tyr^701^ phosphorylation induced by the conditioned media from infected HUVEC (*lane 7*) was reduced when p38 MAPK was inhibited in the donor cells (*lane 8*). Conditioned supernatants from uninfected HUVEC had no effect on STAT1 phosphorylation (*lanes 5* and *6*). These results clearly show that p38 signaling is required for primary expression of IFNs and other STAT1-activating cytokines.

##### IFN Signaling Is Modified by p38 MAPK Activity

**FIGURE 5.**
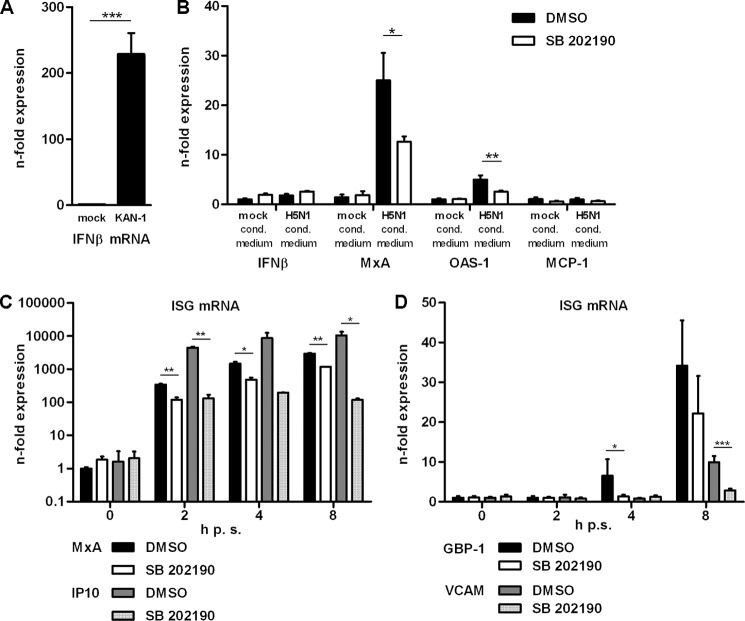
**Influence of p38 MAPK inhibition on cytokine-mediated signaling.** *A* and *B,* H5N1-conditioned medium experiment; donor cells for the production of conditioned medium were mock infected or infected with 5 m.o.i. of KAN-1 for 3 h. Acceptor cells were preincubated with 20 μm SB 202190 or left untreated. UV-inactivated and filtered conditioned media were transferred on acceptor cells for 2 h. Levels of IFNβ mRNA in donor (*A*) and acceptor cells (*B*) as well as ISG mRNAs were detected by qRT-PCR. The mean *n*-fold expression (±S.D.) of one representative experiment normalized to control is depicted. *C* and *D,* HUVEC were preincubated with 20 μm SB 202190 or DMSO. Cells were stimulated with 100 units/ml of recombinant human IFNβ (*C*) or IFNγ (*D*) and post-incubated with SB 202190 for the indicated time points. Expression of ISG mRNAs was analyzed by qRT-PCR. Mean *n*-fold expression (±S.D.) of one representative experiment normalized to control is depicted.

The results so far raised the question as to whether the observed impact of p38 inhibition on secondary ISG expression is simply due to reduced levels of IFNs in the primary response to infection or whether there are additional steps in signaling that are regulated by the kinase. To discriminate between these two scenarios, the consequences of SB 202190-mediated p38 inhibition on the induction of ISG mRNAs were tested after stimulation with conditioned medium. This allows for producing a realistic stimulation with the complete set of IFNs and other cytokines and chemokines that are released upon HPAIV infection. Conditioned media from mock- or KAN-1-infected HUVEC were transferred to SB 202190- or DMSO-pretreated acceptor cells, respectively. qRT-PCR confirmed efficient IFNβ mRNA expression in donor cells (Fig. 5*A*). A potential transfer of infectious particles onto the reporter cells was ruled out by gene-specific qRT-PCR of viral genes. No viral genomic RNA or mRNA of M1 were detectable (data not shown). The reporter cells treated with KAN-1-conditioned medium showed expression of ISGs such as *MX1* or *OAS1*, and the inhibition of p38 MAPK activity led to a significant reduction in mRNA induction (Fig. 5*B*). These observations clearly indicate that besides the impact of p38 on the primary viral gene induction, the kinase additionally controls the induction of IFN- or other STAT-activating cytokine-stimulated gene expression.

To further confirm that p38 really acts on IFN-induced gene expression responses, the effect of p38 inhibition on cells stimulated for 2, 4, or 8 h with IFNβ or -γ in the presence or absence of SB 202190 was examined. Fig. 5*C* shows reduced IFNβ-induced expression of the ISG mRNAs for MxA and IP10 in HUVEC at all time points when p38 signaling was impaired. Interestingly, this was also true for the IFNγ-induced genes *GBP1* and *VCAM1* (Fig. 5*D*). These results were confirmed by promoter analysis of interferon-stimulated genes using the dominant-negative MKK6 mutant thereby ruling out nonspecific off-target effects of the inhibitor (supplemental Fig. S3*B*).

##### MAP Kinase p38 Directly Influences ISG Promoter Activity by the Phosphorylation of STAT1 at Ser^727^

A common mediator of signaling induced by type I and II IFN is STAT1. In the case of IFNβ stimulation, it forms the IFN-stimulated gene factor 3 together with STAT2 and IRF9 and activates transcription from promoters containing interferon-stimulated response elements (ISRE). Upon stimulation with IFNγ, STAT1 homodimers are formed that enhance interferon γ-activated site (GAS)-dependent gene transcription. It has previously been proposed that STAT1 serine phosphorylation caused by a variety of stimuli is sensitive to the inhibition of p38 MAPK (42, 43), but there is also evidence of the existence of a STAT-independent mechanism upon IFN stimulation (44). Although tyrosine phosphorylation of STAT1 constitutes the essential prerequisite for biological activity by triggering DNA binding after nuclear accumulation, phosphorylation at serine 727 seems to be required for full transcriptional activity induced by IFNs (24, 45).

**FIGURE 6.**
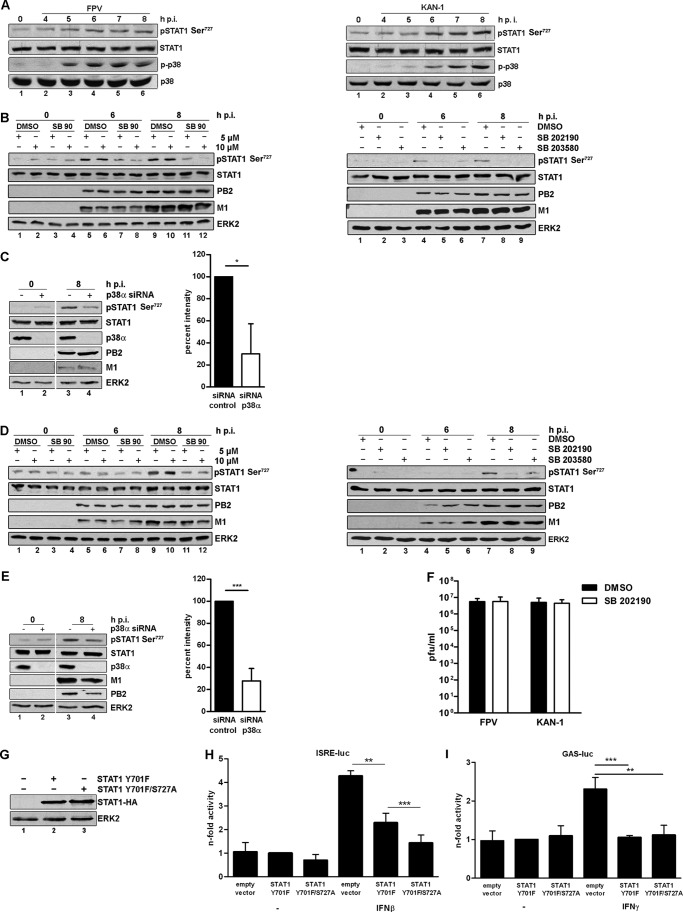
**Influenza A virus infection induces phosphorylation of STAT1 on serine 727.** *A,* Western blot analysis of total lysates of A549 cells infected with 5 m.o.i. of FPV or KAN-1. P-STAT1 Ser^727^ (*upper panel*) and p-p38 (*middle panel*) were detected 4–8 h post-infection. Equal loading was verified by the detection of total STAT1 and total p38. *B* and *D, left:* A549 (*B*) or Vero cells (*D*) were preincubated with 5 or 10 μm SB 202190 in comparison to DMSO. Cells were infected with 5 m.o.i. of FPV and incubated with the respective concentrations of SB 202190 for the indicated time points. *Right,* A549 (*B*) or Vero cells (*D*) were preincubated with 10 μm SB 202190 or SB 203580 in comparison to DMSO. Cells were subsequently infected with FPV (5 m.o.i.) for the indicated time points. *C* and *E, left,* A549 (*C*) or Vero cells (*E*) were transfected with *p38*α-specific siRNA. 48 h post-transfection cells were infected with FPV (5 m.o.i.) for 8 h. Efficient siRNA-mediated knockdown was confirmed by p38α detection. *B–E*, P-STAT1 Ser^727^ (*upper panel*) and viral proteins PB2 and M1 (*middle panels*) were detected by Western blot analysis. Equal loading was verified by the detection of total STAT1 and total ERK2. *C* and *E, right,* densitometric analysis of FPV-induced STAT1 Ser^727^ phosphorylation levels in the presence or absence of *p38*α siRNA. Phosphorylation levels were estimated as the relative intensity of the appropriate phosphorylation bands to the loading control normalized to the appropriate uninfected controls. Intensities are depicted as mean ± S.D. of three independent experiments. *F,* Vero cells were preincubated with 20 μm SB 202190 or DMSO and subsequently infected with FPV (1 m.o.i.) for 9 h. Viral titers are depicted as mean ± S.D. of three independent experiments. *G,* expression of STAT1 Y701F and double mutant (STAT1 Y701F/S727) in A549 cells was confirmed by Western blot analysis (*upper panel*, *lanes 2* and *3*). Equal loading was confirmed by the detection of total ERK2. *A-E* and *G,* blots are representative of three independent experiments. *H* and *I*, A549 cells stably expressing STAT1 Y701F, STAT1 Y701F/S727A, or the empty vector were transfected with the different ISG promoters, as indicated. Cells were stimulated with 500 units/ml of recombinant human IFNβ (*H*, ISRE) or IFNγ (*I*, GAS). 8 h post-stimulation promoter activity was measured and the results are depicted as mean *n*-fold (± S.D.) of three independent experiments normalized to STAT1 Y701F activity.

To study the impact of STAT1 Ser^727^ phosphorylation on the production of ISGs and its dependence on p38 MAPK activity in HPAIV infection, A549 lung epithelial cells were used. These cells allow the efficient transfection of dominant-negative STAT1 constructs that is not possible in HUVEC. A549 showed the same dependence of IFN and ISG induction on functional p38 MAPK signaling as observed in HUVEC (supplemental Figs. S3 and S4*A*) and inhibitory effects of the compound or p38α MAPK knockdown on viral replication in A549 could also be ruled out (supplemental Fig. S4*B*, C). Western blot analysis confirmed that infection with 5 m.o.i. of both H7 (FPV) and H5 (KAN-1) subtype viruses resulted in the activation of p38 (Fig. 6*A*, *middle panels*, *lanes 3* and *4*), which occurred simultaneously with the phosphorylation of STAT1 at Ser^727^ (*upper panels*). To examine whether p38 MAPK activity is required for Ser^727^ phosphorylation, A549 cells were pretreated with 5 or 10 μm SB 202190 for 30 min, infected with FPV (5 m.o.i.), and subsequently incubated with the respective concentrations of SB 202190 for the indicated time points (Fig. 6*B*, *left*). Western blot analysis confirmed reduced STAT1 Ser^727^ phosphorylation (*upper panel*, *lanes 7*, *8*, *11*, and *12*) in a concentration-dependent manner when p38 was inhibited, implying that the kinase is required for serine phosphorylation of STAT1 in influenza virus infection. Efficient infection was confirmed by immunoblotting for viral proteins PB2 and M1 (*middle panels*).

Furthermore, nonspecific off-target effects of SB 202190 on the FPV-induced STAT1 Ser^727^ phosphorylation were ruled out by using a second p38-specific inhibitor, SB 203580 (Fig. 6*B*, *right*, *upper panel*, *lanes 6* and *9*). In addition, the dependence of STAT1 Ser^727^ phosphorylation on a functional p38 pathway was analyzed by molecular means by using *MAPK14*-specific siRNA (Fig. 6*C*, *left*, *upper panel*, *lane 4*), showing a reduction in phosphorylation levels upon knocked down p38α by ∼70% (Fig. 6*C*, *right*).

To rule out any secondary effects of released IFNs on STAT1 phosphorylation at Ser^727^, Vero cells lacking functional type I IFN genes were pretreated with different concentrations of SB 202190 or DMSO and subsequently infected with FPV (5 m.o.i.) for the indicated time points (Fig. 6*D*, *left*). Immunostaining of phosphorylated STAT1 at Ser^727^ clearly demonstrates the dependence of this post-translational modification on functional p38 signaling in the context of HPAIV infection (*upper panel*, *lanes 11* and *12*). Efficient viral propagation was confirmed by immunoblotting for viral proteins PB2 and M1 (*middle panels*). In addition, these findings were confirmed by a second p38-specific inhibitor SB 203580 (Fig. 6*D*, *right*, *upper panel*, *lane 9*) and by an inhibitor independent approach by using *MAPK14*-specific siRNA (Fig. 6*E*, *left*, *upper panel*, *lane 4*). Here, as observed in A549 cells, FPV-induced STAT1 Ser^727^ phosphorylation was reduced by ∼70% when p38α was knocked down (Fig. 6*E*, *right*). Furthermore, an influence of p38 MAPK inhibition on viral replication in Vero cells was ruled out by standard plaque assays (Fig. 6*F*).

To further verify the crucial role of p38-mediated STAT1 Ser^727^ phosphorylation on IFN-induced gene transcription, reporter gene assays in cells that stably express a dominant-negative STAT1 (Y701F) or STAT1 (Y701F/S727A) double mutant were performed. Equal expression levels of the different mutants were confirmed by Western blot analysis of total cell lysates (Fig. 6*G*). Mutant-expressing cells were stimulated with IFNβ (ISRE-luc) or -γ (GAS-luc) for 8 h. Fig. 6*H* shows that stimulation with IFNβ induced ISRE-dependent promoter activity around 4-fold in empty vector-expressing cells. Expression of the dominant-negative STAT1 Y701F mutant decreased promoter activity by 50% and, indeed, expression of the STAT1 double mutant (Y701F/S727A) further reduced IFN-induced promoter activity. In the case of GAS promoter activity, only empty vector-expressing cells showed increased activity after IFNγ stimulation (Fig. 6*I*). Expression of the dominant-negative STAT1 Y701F mutant led to a complete loss of activity that was not further affected by S727A mutation.

##### p38 MAPK Inhibition Leads to the Suppression of Early Innate Immune Responses in Vivo

**FIGURE 7.**
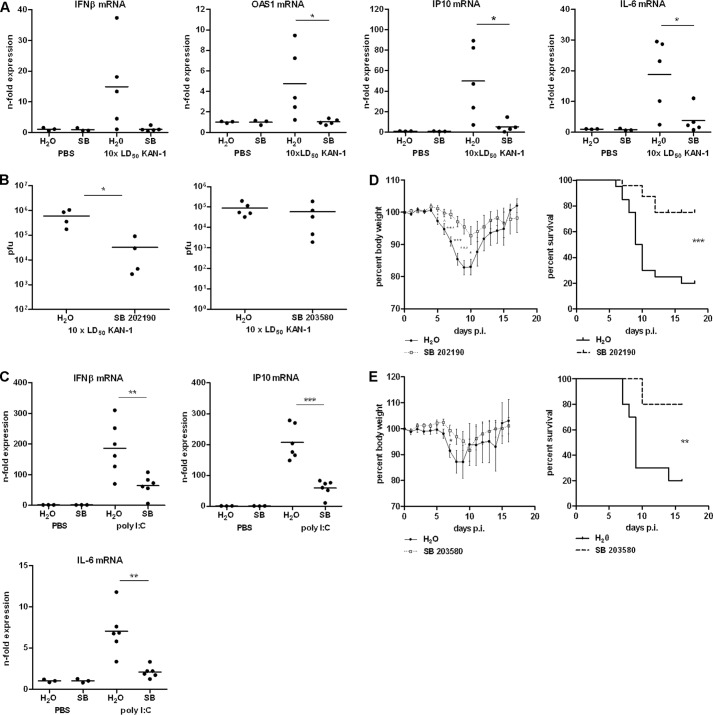
**Effects of p38 inhibition on viral pathogenesis *in vivo*.** BALB/c mice infected with 10 × LD_50_ of KAN-1 were treated with 20 mg/kg/day SB 202190 hydrochloride, SB 203580 hydrochloride, or solvent via intraperitoneal injection directly after infection. *A,* expression changes of different cytokine mRNAs in lungs were analyzed 2 days post-infection by qRT-PCR. *n*-Fold expression in individual animals normalized to uninfected control is depicted. *B,* viral lung titers 2 days post-infection of individual animals are depicted. *C,* BALB/c mice were treated with 20 mg/kg SB 202190 3 h prior to intranasal stimulation with 1 μg of poly(I:C) in PBS. Expression changes of different cytokine mRNAs in lungs were analyzed 6 h post-stimulation by qRT-PCR. *n*-Fold expression in individual animals normalized to PBS-stimulated respective controls is depicted. *D* and *E,* body weight curves; animals were excluded from the analysis when reaching less than 75% of the initial body weight. *D,* mean % body weight of 1–9 (initial group size) animals normalized to initial weight ± S.E. from two independent experiments is depicted. Survival curves; %-survival of 1–9 (initial group size) animals from two independent experiments is depicted. *E,* mean % body weight of 1–10 (initial group size) animals normalized to initial weight ± S.E. is depicted. Survival curves; %-survival of 1–10 (initial group size) is depicted.

The induced cytokine storm during severe influenza infections leads to major morbidity and mortality. A significant association between excessive early cytokine response, immune cell recruitment, and poor outcome has been documented for avian H5N1 infection (2). To investigate the role of p38 MAPK signaling in the dysregulation of cytokine expression after infection with a human pathogenic H5N1 isolate *in vivo*, BALB/c mice were infected with 10 × LD_50_ KAN-1 that had never been passaged in mice. Infection of BALB/c with this strain causes severe disease, resulting in neurological deficits and high mortality rates. Directly after infection, mice were treated intraperitoneally with 20 mg/kg of water-soluble SB 202190 hydrochloride or vehicle once per day. Two days post-infection, lungs from mice were extracted and total RNA was isolated for qRT-PCR. Fig. 7*A* shows lung IFNβ mRNA levels from PBS-treated control mice in comparison to KAN-1-infected mice. The KAN-1-induced expression of IFNβ mRNA observed in vehicle-treated mice was nearly completely abolished in the presence of the p38 inhibitor SB 202190. This was also true for different ISGs such as *OAS1* and *IP-10*. Interestingly, the transcription of NF-κB-dependent gene *IL6* was also significantly suppressed after p38 MAPK blockade (*right*). These results impressively illustrate the importance of p38 in the induction of the cytokine storm *in vivo*. To verify whether the observed suppression of cytokine expression after p38 inhibition occurs due to alterations in viral propagation, BALB/c mice were infected with 10 × LD_50_ KAN-1 and treated with SB 202190 hydrochloride or vehicle directly after infection, as described above. Two days post-infection, lungs from mice were extracted and viral lung titers were determined via standard plaque titration. Following SB 202190 treatment, viral titers were decreased by ∼10-fold compared with the lung titers of vehicle-treated mice (Fig. 7*B*, *left*). Interestingly, these effects on viral replication were not observed upon treatment with another p38 inhibitor (SB 203580 hydrochloride, Fig. 7*B*, *right*), whereas reduced cytokine expression levels upon KAN-1 infection could be observed, although less pronounced compared with SB 202190 treatment (supplemental Fig. S5).

Considering a possible attenuated viral propagation after p38 inhibition, a decrease in cytokine expression would not be surprising. Thus, cytokine induction was determined in a replication-independent system using the double-stranded RNA analog poly(I:C). BALB/c mice were treated intraperitoneally with 20 mg/kg SB 202190 hydrochloride or vehicle 3 h prior to intranasal stimulation with 1 μg poly(I:C). Lungs from mice were extracted 6 h post-stimulation and total RNA was isolated for qRT-PCR. Fig. 7*C* shows the lung cytokine mRNA levels from PBS-treated control mice in comparison to poly(I:C)-stimulated mice. The repression of dsRNA-induced responses in the lungs of mice that were treated with SB 202190 (Fig. 7*C*) reflects the direct function of p38 MAP kinase in the induction of excessive cytokine and chemokine production.

Dysregulation of the innate cytokine response indicates disease severity and death during HPAIV infection (1, 2). To analyze whether blunting the expression of cytokines using the p38 inhibitor SB 202190 could protect mice from lethal influenza infection, BALB/c mice were infected with 10 × LD_50_ of KAN-1 and treated intraperitoneally with SB 202190 hydrochloride or vehicle, as described earlier. Fig. 7*D* shows the body weight and survival curves of KAN-1-infected mice treated with SB 202190 in comparison to vehicle-treated mice. Although vehicle-treated mice lost body weight as early as 5 days post-infection, weight loss in SB 202190-treated mice was delayed and was detectable 8 days post-infection. Both experimental groups showed peaks in weight loss 10 days post-infection, with more pronounced effects in control mice. This is supported by the survival curves that show enhanced survival of SB 202190-treated mice. Only one-third of these mice died, whereas nearly 85% mortality was observed in vehicle-treated mice. Furthermore, these results on survival proportions could be fully confirmed with the p38-specific inhibitor SB 203580 hydrochloride (Fig. 7*E*). Considering the fact that viral propagation was not affected by SB 203580 treatment, these findings demonstrate that early suppression of cytokine amplification by inhibiting p38 MAP kinase activity significantly protects mice from lethal influenza virus infection and impressively emphasizes the important role of the cytokine storm in the viral pathogenicity of HPAIV.

## DISCUSSION

Human influenza virus infections caused by highly virulent H5N1 variants are associated with high mortality rates that clearly emphasize the need for further knowledge about the biologic characteristics of these infections to identify new targets for antiviral interventions. It has been shown that HPAI viruses induce generalized infections associated with an overwhelming production of cytokines and chemokines that has been hypothesized to contribute to viral pathogenesis (1, 2). Endothelial cells have been shown to play a major role in cytokine dysregulation (6, 7, 9). Furthermore, innate immune cell recruitment and early innate cytokine expression seem to be independent events with endothelial cells at the center of both processes (9), possibly repositioning the role of immune cells in this context to secondary importance. So far, different transcription factors like IRF3, NF-κB, AP1, and NFATC4 have been shown to be involved in H5N1-induced hyperactivation of the innate immune system, with their significance depending on the cell type used (6–8). Furthermore, it is well known that p38 MAP kinase represents an important factor in various inflammatory diseases, modulating the actions of the aforementioned transcription factors upon different stimuli in different cell types (38–40). The aim of this study was to analyze the impact of p38 MAP kinase on HPAIV-induced cytokine induction in primary endothelial cells. Furthermore, the role of the kinase in H5N1 pathogenicity was analyzed *in vivo*, identifying p38 MAPK as a likely target for antiviral intervention for the first time.

Global mRNA profiling confirmed that upon infection of primary endothelial cells with a highly pathogenic influenza A virus of the H7N7 subtype, p38 had an excessive impact on the FPV-induced transcriptome. By mathematical calculation, nearly all HPAIV-induced genes were either partially (23%) or fully (71%) dependent on this pathway; the majority of these mRNAs belong to the immune/inflammatory response and chemotaxis gene ontology categories. These findings indicate a pivotal role of p38 MAPK in HPAIV-induced cytokine and chemokine dysregulation. Furthermore, this suggests a function for this signaling pathway in immune cell recruitment upon influenza infection by regulating the expression of a number of chemotactic cytokines such as CXCL9, -10, -11, and CCL5 by endothelial cells; these cells are of primary importance for innate immune cell recruitment (9). Additional experimental approaches are required to reveal whether p38 MAPK is critical for chemokine expression and subsequent immune cell recruitment upon HPAIV infection *in vivo*, as has been shown for enteric bacterial infection of the colonic mucosa (46).

Interestingly, IFNβ mRNA was found among the FPV-induced mRNAs that were switched off in the presence of the p38 inhibitor SB 202190, highlighting the obligatory dependence of HPAIV-induced IFNβ production on functional p38 signaling. So far, IRF3 and NF-κB have been shown to be essential for activation of the IFNβ promoter in H5N1-infected endothelial cells (6, 7). Although IRFs are also the most abundant transcription factors modulating the FPV-induced transcriptome, as determined by promoter analysis, IFNβ induction could not be confirmed to be NF-κB-dependent upon H7N7 infection (7). In contrast, the present study shows that p38 MAPK activity is needed for both H7N7- and H5N1-induced IFNβ expression, indicating a more global role of p38 signaling in cytokine induction provoked by HPAIV infection. This was confirmed by infection studies and stimulation with total RNA from FPV- or KAN-1-infected cells, respectively. Similar results were obtained in alveolar epithelial cells, indicating a general impact of p38 signaling on HPAIV-induced IFNβ production that is not cell type specific. This supports previous results from Hui and colleagues (8) obtained in primary human macrophages.

Besides a direct impact on the IFNβ promoter that could be verified experimentally, not only by inhibitor studies but also by the overexpression of a dominant-negative MKK6 mutant acting upstream of p38 MAPK, induction of HPAIV-induced ISGs was decreased upon p38 inhibition and siRNA-mediated knockdown. Furthermore, all genes previously described to be ISGs were found among the p38-regulated genes. Stimulation with type I and type II IFN or virus-conditioned media revealed a direct dependence of ISG expression on p38 MAPK activity in endothelial cells. In addition to the aforementioned IRF transcription factors, promoter analysis of the FPV-induced transcriptome identified the ISRE consensus motif to be overrepresented within the group of up-regulated genes (7). One of the major factors needed for type I and type II IFN signaling is STAT1. Upon activation, STAT1 participates in gene induction via ISRE and GAS consensus motifs, thereby inducing the transcription of ISGs. One possibility for p38-dependent enhancement of ISG expression is the direct phosphorylation of serine 727 within the STAT1 protein. This phosphorylation site has been shown to be important for full transcriptional activity induced by IFNs (24, 45). Although the role of p38 MAPK signaling in Ser^727^ phosphorylation upon IFN signaling is still controversially discussed, the present study clearly shows for the first time the obligatory dependence of influenza virus-induced Ser^727^ phosphorylation of STAT1 upon functional p38 MAPK signaling. Such a tyrosine phosphorylation-independent Ser^727^ phosphorylation step has been observed upon stimulation by different stress stimuli and was shown to be important for transcriptional effects that are independent of STAT1 binding to DNA. Therefore, it is likely that STAT1 can function as a transcriptional co-activator by interacting with DNA-bound factors (47), thereby modulating influenza A virus-induced cytokine and chemokine expression.

Several studies have suggested that the STAT2 transactivating domain provides IFN-stimulated gene factor 3 transactivation function asserting that the STAT1 transactivating domain and its phosphorylation at serine 727 are not essential for type I IFN-induced transcriptional activity (48, 49). Here, a more recent report showing a beneficial impact of Ser^727^ phosphorylation on type I IFN-induced transcription could be confirmed by promoter studies using STAT1 Y701F and the Y701F/S727A double mutant (45).

Dysregulation of early cytokine induction in HPAIV infection appears to determine disease severity by enhancing viral pathogenicity (1, 2). The same cytokines that orchestrate the infiltration of immune cells, resulting in phagocytosis and intracellular killing of the pathogen and the control of infection, are responsible for tissue remodeling and organ damage when produced in excessive amounts. Consequently, although their primary function is to protect the host and repair tissue when injured, these cytokines are mediators of disease and thus are targets for anti-inflammatory therapy (50). Inhibition of p38 MAP kinase *in vivo* by intraperitoneal treatment with the chemical compound SB 202190 clearly demonstrated that H5N1-induced cytokine hyperactivation was nearly completely abolished and this impairment was independent of reduced viral replication, as shown by stimulation with the dsRNA analog poly(I:C). A major drawback of anti-inflammatory therapy against infections is a reduction in the antiviral host gene response, allowing the pathogen to propagate unhindered, thereby supporting further spread. In contrast, SB 202190-mediated inhibition of p38 MAPK signaling *in vivo* even led to reduced viral propagation, indicating the presence of a virus-supportive function for p38 in influenza A virus-infected animals. Previously, it was hypothesized that virus internalization is impaired by p38 MAPK inhibition due to reduced early endosome antigen 1 (EEA1) phosphorylation upon TLR4-MyD88 signaling, which has been described to enhance endocytosis (35). Furthermore, the retention of viral ribonucleoprotein complexes in the nucleus was observed with defective MAPK p38 signaling and linked to reduced phosphorylation of viral nucleoprotein (34). Although all involved proteins are expressed in HUVEC, reduced viral replication was not observable. Similar results were obtained in A549 cells, even though all the proteins concerned were expressed. Whether these mechanisms described *in vitro* might be the reasons for impaired viral replication *in vivo* needs to be further analyzed.

In conclusion, the present study reveals for the first time that inhibition of the p38 MAPK pathway significantly protects mice from lethal H5N1 infection. Furthermore, an overall virus-supportive function of p38 MAP kinase was confirmed in infected animals, although considerable levels of ongoing viral replication were still observed. These findings demonstrate that early suppression of cytokine amplification by inhibiting p38 activity significantly leads to protection of mice from lethal influenza virus infection and impressively emphasizes the important role of the cytokine storm in the viral pathogenicity of HPAIV. Targeting p38 MAP kinase might thus be a promising approach for antiviral intervention.

## Footnotes

4 The abbreviations used are:

HPAIV: highly pathogenic avian influenza virus
AP1: activator protein 1
FC: fold-change
FPV: fowl plague virus A/FPV/Bratislava/79 (H7N7)
GAS: interferon γ-activated site
HUVEC: human umbilical vein endothelial cells
ISG: interferon-stimulated gene
IRF: interferon regulatory factor
ISRE: interferon-stimulated response element
KAN-1: A/Thailand/KAN-1/04 (H5N1)
MKK: mitogen-activated protein kinase kinase
m.o.i.: multiplicity of infection
NF-κB: nuclear factor κ-light chain enhancer of activated B-cells
qRT: quantitative real-time
ISG: interferon-stimulated gene
DMSO: dimethyl sulfoxide.
